# Hydrogen Storage Performance of Mg/MgH_2_ and Its Improvement Measures: Research Progress and Trends

**DOI:** 10.3390/ma16041587

**Published:** 2023-02-14

**Authors:** Xinglin Yang, Wenxuan Li, Jiaqi Zhang, Quanhui Hou

**Affiliations:** 1 School of Energy and Power, Jiangsu University of Science and Technology, Zhenjiang 212003, China; 2 School of Automotive Engineering, Yancheng Institute of Technology, Yancheng 224051, China

**Keywords:** Mg/MgH_2_, hydrogen storage performance, improvement measures, catalyst

## Abstract

Due to its high hydrogen storage efficiency and safety, Mg/MgH_2_ stands out from many solid hydrogen storage materials and is considered as one of the most promising solid hydrogen storage materials. However, thermodynamic/kinetic deficiencies of the performance of Mg/MgH_2_ limit its practical applications for which a series of improvements have been carried out by scholars. This paper summarizes, analyzes and organizes the current research status of the hydrogen storage performance of Mg/MgH_2_ and its improvement measures, discusses in detail the hot studies on improving the hydrogen storage performance of Mg/MgH_2_ (improvement measures, such as alloying treatment, nano-treatment and catalyst doping), and focuses on the discussion and in-depth analysis of the catalytic effects and mechanisms of various metal-based catalysts on the kinetic and cyclic performance of Mg/MgH_2_. Finally, the challenges and opportunities faced by Mg/MgH_2_ are discussed, and strategies to improve its hydrogen storage performance are proposed to provide ideas and help for the next research in Mg/MgH_2_ and the whole field of hydrogen storage.

## 1. Introduction

**Figure 1:**
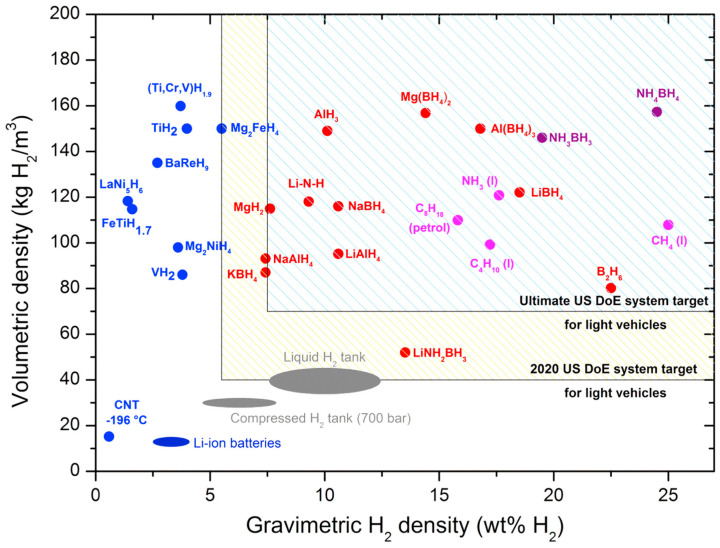
Overview of different hydrogen storage systems and their volumetric and gravimetric hydrogen density. The U.S. Department of Energy targets for the hydrogen storage system are also shown for comparison. Copyright 2017, Elsevier. Reproduced with permission from [7].

Currently, hydrogen storage technologies can be divided into two categories: physical storage and chemical storage. The former includes liquefied hydrogen storage, compressed gas storage, cryo-compression and hydrogen storage by solid-state physisorption materials. The latter includes hydrogen storage by solid or liquid state chemisorption materials [1,2,3]. Among all hydrogen storage technologies, solid-state hydrogen storage technology has received a lot of attention, because it not only offers high safety but also high hydrogen storage density [4]. In the past, scholars have studied a variety of solid-state hydrogen storage materials, including physisorption materials, such as carbon-based materials, metal organic framework (MOFs) and zeolites, as well as chemisorption materials, such as hydrogen storage alloys, complex metal hydrides and lightweight binary metal hydrides. The U.S. Department of Energy (DOE) specifies that the on-board hydrogen storage system should have a hydrogen storage capacity of 5.5 wt.% at a hydrogen pressure of 5–12 bar and a temperature of about 85 °C, and the number of cycles of hydrogen storage materials should not be less than 1000 [5,6]. Figure 1 shows not only the different hydrogen storage technologies and their hydrogen storage capacities but also DOE’s goals for hydrogen storage systems for comparison [7]. In general, volumetric density (kg H_2_/m^3^) and gravimetric H_2_ density (wt.%) are commonly used to measure the hydrogen storage capacity of a hydrogen storage system, the former being the mass of hydrogen stored per unit volume of the system, and the latter being the ratio of the mass of hydrogen stored in the system to the mass of the system. However, no hydrogen storage material has been found so far that meets all the requirements proposed by DOE. Therefore, the research of high-capacity and high-performance hydrogen storage materials is the key and the most challenging aspect of the large-scale application of solid-state hydrogen storage technology.

Physisorption materials, such as carbon materials, MOFs and zeolites, rely on weak Vander Waals forces, electrostatic, orbital interactions and other weaker effects for hydrogen adsorption [8,9]. Although the gravimetric H_2_ density (wt.%) DOE requirements are met by some materials, such as activated carbon (~9.0 wt.%) [10,11], microporous porous carbon (~11.2 wt.%) [12] and MOFs (~10.6 wt.%) [13], these materials are difficult to be widely used because of the low binding energy (4–10 kJ/mol) between hydrogen molecules and the material, which leads to the limitation of the hydrogen storage capacity at room temperature and makes it difficult to hold the hydrogen to the surface [3]. Although modification of such hydrogen storage materials with metals can enhance the binding energy, it may tend to create discrete metallic nanoclusters in the materials, thereby hindering their hydrogen storage capacity [2,14,15].

In contrast, chemisorption materials, such as metal hydrides, have strong binding energy (40–80 kJ/mol) between hydrogen atoms and the material, which helps hydrogen storage materials to store hydrogen under ambient conditions [3]. The hydrogen storage capacity, thermodynamic/kinetic performance and cycling performance of metal hydrides (e.g., hydrogen storage alloys, complex metal hydrides, light binary metal hydrides, etc.) have received a lot of attention.

There are abundant types of hydrogen storage alloys, including AB_5_, AB, AB_2_, A_2_B, AB_3_ alloys, etc. In fact, catalyst doping and partial substitution of A or B by metallic elements are widely used to improve the hydrogen storage performance of hydrogen storage alloys. As early as 1976, Bronoel et al. [16] reported the hydrogen storage capacity of LaNi_5_ of type AB_5_. Later studies showed that LaNi_5_ had potential exploitability [17,18,19]. Singh et al. [18] found that the doping of graphite enhanced the hydrogen storage performance of LaNi_5_, especially the reversibility. In addition to the introduction of catalysts, Dashbabu et al. [19] found some improvement in the hydrogen storage performance of LaNi_5_ after partial substitution of Ni by Al, and the degree of modification was influenced by the amount of aluminum added. More efforts are needed to enhance the performance of hydrogen storage alloys and make them applicable to a wider range of fields.

Similar to hydrogen storage alloys, complex metal hydrides are also a “family”, which includes alanates, borohydrides, nitrides, etc. [2,20,21]. Complex metal hydrides have considerable hydrogen storage capacity, such as LiBH_4_ with a theoretical hydrogen storage capacity of 18.4 wt.% [22]. Nevertheless, the insufficient thermodynamic/kinetic performance and irreversibility of such hydrogen storage materials limit their practical applications [23,24,25]. In response, scholars have taken measures, such as catalyst doping, to improve them. Different catalysts have emerged, including metal-based catalysts [26,27,28,29,30] and nonmetallic catalysts [31,32]. Recently, Ismail et al. [26] found that the initial dehydrogenation temperature of NaAlH_4_ was reduced by about 100 °C after the introduction of CoFe_2_O_4_. In addition, Li et al. [33] found that composites formed by NaAlH_4_ with nanoporous material (Raney nickel) reduced the initial dehydrogenation temperature by about 125 °C compared to pure NaAlH_4_. 

MgH_2_, a lightweight binary metal hydride, has been widely investigated as one of the most promising solid hydrogen storage materials due to its low cost, abundant resources, high hydrogen storage capacity and good reversibility. The reversible hydrogen storage capacity of Mg/MgH_2_ can reach approximately 7.6 wt.%, which satisfies DOE’s regulations [1,5,6,34]. However, the hydrogen storage performance of Mg/MgH_2_ has certain drawbacks, such as high thermodynamic stability (enthalpy ~76 kJ/mol and entropy ~130 kJ/mol [35]), slow hydrogen absorption/desorption kinetics and high temperature of hydrogen absorption/desorption, which are the main reasons why Mg/MgH_2_ is difficult to be used on a large scale. Under atmospheric pressure, the dehydrogenation temperature of MgH_2_ is over 300 °C; the temperature required for the reaction of magnesium with hydrogen to form MgH_2_ exceeds 300 °C when the hydrogen pressure exceeds 3 MPa. In addition, the chemical bond of MgH_2_ (Mg-H) is too stable resulting in an activation energy (Ea) of about 160 kJ/mol for the dehydrogenation reaction of MgH_2_. Excitingly, due to the good exploitability of Mg/MgH_2_, scholars have been able to significantly improve its hydrogen storage performance through a series of measures, mainly including alloying treatment, nano-treatment and catalyst doping [34,35,36,37]. Alloying treatment and nanosizing treatment can effectively reduce the thermodynamic stability of Mg/MgH_2_, while both have very limited contribution to the kinetic performance of Mg/MgH_2_. Catalyst doping, with its relatively powerful modification ability, is the best option used to compensate for the lack of kinetic performance of Mg/MgH_2_ and, therefore, has received wide attention as one of the most convenient and feasible methods to improve the kinetics of Mg/MgH_2_.

In this paper, alloying treatment, nano-treatment and catalyst doping measures to improve the hydrogen storage performance of Mg/MgH_2_ are discussed in detail, and the catalytic performance and mechanism of various metal-based catalysts are discussed and analyzed in depth. Finally, the challenges and opportunities faced by Mg/MgH_2_ are discussed, and strategies to improve its hydrogen storage performance are proposed to provide ideas and help for the next research in Mg/MgH_2_ and the whole field of hydrogen storage. 

## 2. Improvement of Hydrogen Storage Performance of Mg/MgH_2_ by Alloying Treatment

It is well known that the high stability of Mg/MgH_2_ originates from the bond strength between the Mg-H bonds, which can be effectively mitigated by alloying treatments, due to the structural and compositional adjustments that destabilize Mg/MgH_2_ [38]. In general, the alloying treatment means that additional metallic elements are used to form new alloys with Mg elements. The alloying treatment based on Mg/MgH_2_ and its improvement of hydrogen storage performance are summarized in Table 1.

Alloying treatment, not only for the modification of alloying materials, is also helpful for the modification of Mg/MgH_2_. Back in 2011, Mg_0.95_In_0.05_ solid solution, was found to have a smaller enthalpy of hydrogen absorption compared to pure Mg, which was about 68.1 kJ/mol [47]. Similarly, later, alloy materials, such as 0.75Mg-0.25Ti and 0.75Mg-0.25V were also found to have reduced enthalpy of hydrogen absorption/dehydrogenation of Mg/MgH_2_ [39]. Recently, Lu et al. [48] explored the hydrogen storage performance of Mg_x_Mn_1−x_ and found that the introduction of Mn elements destabilized Mg/MgH_2_. At higher Mn content, the thermodynamic destabilization of Mg/MgH_2_ makes it reversible for hydrogen absorption and dehydrogenation even at room temperature. Furthermore, Khan et al. [40] found that the Mg_2_Ni alloy had a low enthalpy of hydrogen absorption (−57.47 kJ/mol) and dehydrogenation (61.26 kJ/mol). The thermodynamic performance of the Mg/MgH_2_ was improved; however, there was a considerable loss of hydrogen storage capacity, with a maximum storage capacity of only about 3.44 wt.%. As early as the 1980s, Mg_2_Ni and Mg_2_Cu have been documented [49,50]. Not only that, many metallic elements were found to form different types of alloys with Mg. Recent studies have shown that metals such as Al [41], Ce [42], Pr [43] and Gd [44] could also be alloyed with Mg and the resulting alloys possessed good hydrogen storage performance. 

Passing et al. [41] found that Mg-Al alloys possessed good thermodynamic/kinetic performance and at the same time, the alloy could store about 5.8 wt.% of hydrogen. However, Mg_90_Ce_3_Ni_7_ alloy [42], Pr-Mg-Ni-based alloy [43] and Gd_5_Mg_95-x_Ni_x_ type alloy [44] did not exhibit good hydrogen storage capacity. Notably, the hydrogen absorption and desorption performance of these hydrogen storage materials were enhanced due to the addition of different metallic elements. Song et al. [42] found that Mg_90_Ce_3_Ni_7_ alloy had good hydrogen absorption performance at low temperature. The alloy could absorb more than 3.5 wt.% H_2_ within 30 min at 100 °C. In addition, Bu et al. [43] found that the hydrogen diffusion capacity of Pr-Mg-Ni-based alloys increased with the increase of Ni content. However, at 200 °C the hydrogen absorption capacity of the alloys decreased from 5.41 wt.% of Pr_5_Mg_90_Ni_5_ to 4.49 wt.% of Pr_5_Mg_80_Ni_15_ within 60 min. Bu’s team [44] also found that the hydrogen absorption capacity of Gd_5_Mg_95-x_Ni_x_-type alloys decreased with the increase of Ni content, while the hydrogen absorption rate was affected by temperature. Among them, Gd_5_Mg_80_Ni_15_ had the fastest dehydrogenation rate and smaller dehydrogenation activation energy. In addition, the thermomechanical performance of the alloy due to the addition of Gd and the variation of Ni content was superior compared to those of pure Mg. It may not be a coincidence that the higher hydrogen storage capacity of Mg-Al alloys is due to the fact that Mg-Y-Zn [45] and Mg-Ni-Y [46] alloys were also found to have high hydrogen storage capacity.

These specific examples mentioned above demonstrate that alloying treatment is an effective means used to improve the hydrogen storage performance of Mg/MgH_2_ in different ways. However, some of these examples also exemplify the very important point that alloying treatments can effectively alleviate the thermodynamic performance of Mg/MgH_2_ but tend to lead to the sacrifice of some of the considerable hydrogen storage capacity of the hydrogen storage material. Therefore, there is still room for further development of alloying, and in order to achieve better improvement, it is necessary to optimize the alloy preparation technology and control the type and content of doping elements, etc.

## 3. Improvement of Hydrogen Storage Performance of Mg/MgH_2_ by Nanosizing 

Nanosizing treatment means reducing the particle size or the crystallite size of Mg/MgH_2_ down to the nanometer level. As nanosizing usually allows Mg/MgH_2_ to have small size and great activity, it therefore enhances its thermodynamic/kinetic performance. The commonly used nanomaterial preparation methods can be divided into direct preparation, which mainly includes high energy milling, gas-phase reaction, chemical reduction, etc., and non-direct preparation, which is to limit the particle or crystallite size of Mg/MgH_2_ by scaffolding materials. Among them, high energy milling is traditional and simple, which has been favored by many scholars. Examples of successful nanosizing of Mg/MgH_2_ systems has been summarized in Table 2.

Scholars have prepared Mg/MgH_2_ with different particle or crystallite sizes by ball milling and found that the dehydrogenation temperature decreased as the particle or crystallite size decreased. In 2006, Varin et al. [51] prepared MgH_2_ with particle sizes in the range of 500–600 nm by ball milling and found that peak hydrogen desorption temperature was effectively reduced by about 40–60 °C (from about 414 °C to about 370 °C). In fact, this is not really a nanoparticle (truly nanometric size is about 100 nm or below), but the study showed that the reduction in particle size contributed to the reduction of the hydrogen desorption temperature of MgH_2_. Notably, increasing the amount of γ-MgH_2_ might effectively reduce the hydrogen desorption temperature of MgH_2_. Although, the simple and convenient ball milling method to prepare nanoscale MgH_2_ effectively alleviated the barrier of material dehydrogenation, the size of nanomaterials prepared by this method was still not small enough and prone to agglomeration and impurity incorporation [57]. In order to obtain smaller nanoparticles or crystallites, Zhang et al. [58] prepared Mg particles of about 40 nm using (gas-phase reaction method) an acetylene plasma metal reaction. It was shown that the treated nanomaterials possessed low activation energies for hydrogen absorption and dehydrogenation, 61.6 kJ /mol and 114 kJ/ mol, respectively. In the same year, Norberg et al. [53] prepared three different sizes of Mg nanocrystals using a chemical reduction method. It was shown that the kinetics of hydrogen absorption increased with decreasing size of the nanocrystals. This huge rate enhancement was not only due to the reduction in particle size but was likely due to an increase in the defect density present in smaller nanocrystals. Later studies focused on reducing the particle or crystallite size, in which Liu et al. [58] found that Mg/MgH_2_ particles with sizes of about 8 nm and 25 nm, respectively, prepared by the same chemical reduction method had better kinetic/thermodynamic performance. It is noteworthy that the nanomaterials prepared by the above method are prone to particle aggregation and lack cyclic stability [59]. Therefore, both smaller size nanomaterials and the control of the particle aggregation phenomenon should be of concern, so attaching nanomaterials to a suitable carrier is a good option.

**Figure 2:**
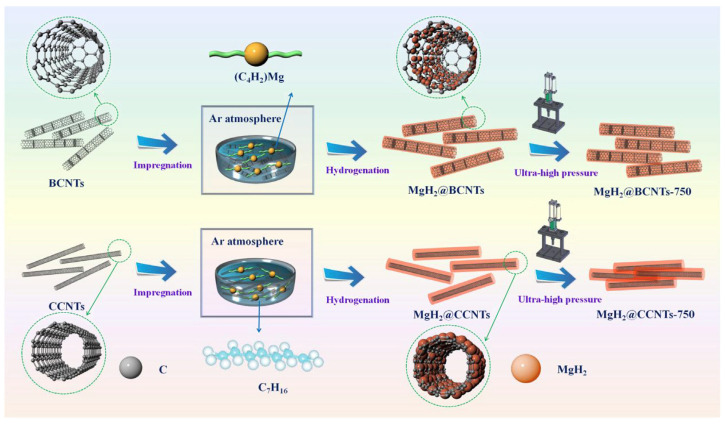
Schematic illustration of the self-assembly of MgH_2_ NPs on the BCNTs and CCNTs by impregnation and hydrogenation and densification under ultra-high pressure of 750 MPa. Copyright 2019, Elsevier. Reproduced with permission from [55].

In order to obtain Mg/MgH_2_ with smaller particle or crystallite size and better hydrogen absorption/dehydrogenation cycling stability, the size of Mg/MgH_2_ can be limited to the nanometer level by inert support materials. In recent years, scaffold materials, such as mesoporous materials, carbon nanotubes and porous carbon materials, have received a lot of attention. As early as 2007, Jongh et al. [60] used porous carbon materials for the first time to constrain magnesium melt so as to prepare nanomaterials. This study prepared magnesium nanocrystals in the range of 2–5 nm or even smaller by controlling the pore size of the carbon material. In a later study, Au et al. [54] used carbon aerogels to load MgH_2_ nanoparticles in the range of 6–20 nm. It was observed that the carbon material as a carrier effectively inhibited the growth of nanoparticles, thus promoting the cycling performance of the nanomaterials. An experiment by Liu et al. [55] on nanoscale MgH_2_ encapsulated with carbon nanotubes demonstrated again the good binding of the scaffold material to MgH_2_, which was prepared by the route shown in Figure 2. The study showed that the hydrogen desorption rate of carbon nanotube-supported nano-MgH_2_ at 275 °C and within 1 h was rapid, and the dehydrogenation amount reached 5.70 wt.%. In addition, the carbon material as a carrier effectively improved the cycling performance of MgH_2_. Recently, Zhang et al. [56] explored the hydrogen storage performance of MgH_2_ supported by different carbon materials, including coconut shell carbon (CSC), multi-walled carbon nanotubes (CNT), graphite (G) and activated carbon (AC). It was shown that all these different types of carbon materials led to different degrees of improvement in the kinetic performance of dehydrogenation/rehydrogenation of Mg/MgH_2_. Among them, CSC as a template can effectively inhibit the growth and agglomeration of MgH_2_. In addition, the layered structure of carbon materials helps to maintain the high specific surface area and high dispersion of nano-Mg/MgH_2_, which leads to the continuous improvement of its hydrogen absorption and desorption performance.

Nanosizing techniques, which improve the thermodynamic/kinetic performance of Mg/MgH_2_ by reducing its particle or crystallite size, are still limited for the improvement of kinetic and cycling performance, and there is still room for development. 

## 4. Improvement of Hydrogen Storage Performance of Mg/MgH_2_ by Catalyst Doping

Catalyst doping can effectively alleviate the dehydrogenation/rehydrogenation energy barrier of Mg/MgH_2_ and improve the kinetic performance of Mg/MgH_2_. A large number of different types of catalysts have been investigated by scholars. It is noteworthy that many nanocatalysts have better catalytic effects because they provide more active catalytic sites and closer contact with Mg/MgH_2_.

In the late 1990s, some scholars used transition metals to improve the kinetics of MgH_2_. In 1999, Liang et al. [61] found that MgH_2_ started to dehydrogenate at a temperature of 200 °C due to the introduction of 5 at.%V, followed by rehydrogenation at a high rate at room temperature. In the same year, Liang’s group [62] also found that Ti, V, Mn, Fe and Ni metal catalysts all showed good catalytic performance for the hydrogen absorption and desorption process of Mg/MgH_2_. Among them, V showed the most significant catalytic effect on the rate of dehydrogenation of MgH_2_, while Ti was the most effective in promoting its hydrogen absorption kinetics. In addition, the activation energy of MgH_2_ dehydrogenation was significantly decreased by the nanosized catalyst. Later, the good catalytic effect of metallic elements, such as Ni, Ti and Fe for MgH_2_ was investigated again [63]. Moreover, this study also showed that the effect of Ti, Fe and Ni co-catalysis would be better, which resulted in a 35.71 kJ/mol decrease in the dehydrogenation activation energy of MgH_2_.

In the next chapters, based on several widely used metal elements, the catalytic performance and mechanism of these metal-based catalysts are discussed and analyzed in detail. Monometals, metal alloys, compounds and metal-based composites formed by transition metal elements, such as nickel (Ni), iron (Fe), titanium (Ti), vanadium (V) and manganese (Mn) in the past ten years, are mainly discussed and analyzed, organized and summarized in Tables 3–7. Among them, the composites are dominated by carbon-supported metal-based catalysts. In addition, many other metals are also mentioned in these metal-based catalysts, such as niobium (Nb), cobalt (Co), zirconium (Zr), etc. 

### 4.1. Nickel (Ni)-Based Catalysts

For a long time, nickel (Ni)-based catalysts have been studied to improve the hydrogen storage performance of Mg/MgH_2_; Table 3 summarizes the improvement of the hydrogen storage performance of Mg/MgH_2_ by nickel-based catalysts in the past decade.

**Figure 3:**
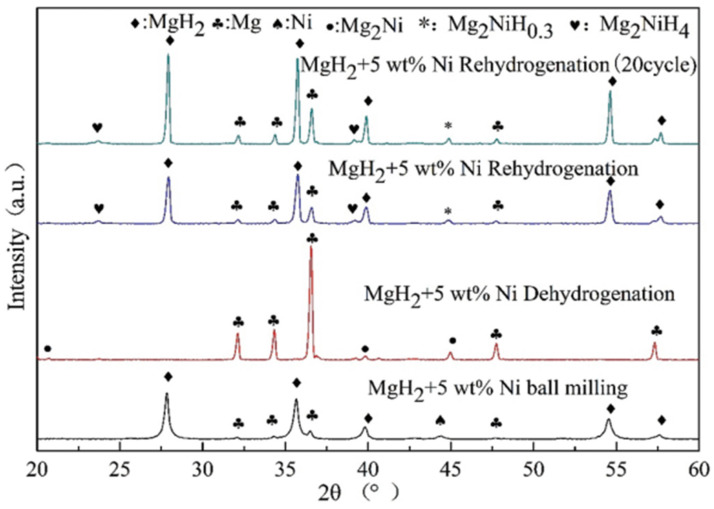
XRD patterns after ball milling, the hydrogen dehydrogenation, rehydrogenation and after the 20th cycle for MgH_2_ + 5 wt.% Ni. Copyright 2021, The Royal Society of Chemistry. Reproduced with permission from [64].

In 2013, it was shown that Ni doping not only improved the dehydrogenation ability of MgH_2_ but also enhanced its rehydrogenation kinetics [63]. Later, El-Eskandarany et al. [78] found that the ball milling time affected the catalytic effect. At 50 °C within 300 min, the sample milled for 25 h absorbed about 3.9 wt.% of H_2_, while the sample milled for 50 h absorbed only 3.6 wt.% of H_2_. Compared to the former, the considerable decrease in hydrogen storage capacity of the latter was due to its higher Ni concentration. Recently, Yang et al. [64] demonstrated that the flaky nickel nanocatalysts could effectively improve the kinetic performance of MgH_2_. However, the cycling performance of MgH_2_ was not enhanced, and there was a significant weakening of the hydrogen storage capacity within 10 cycles. It is worth mentioning that the formation of Mg_2_Ni/Mg_2_NiH_4_ on the surface of Mg/MgH_2_ in the hydrogen absorption and dehydrogenation reaction is an important factor in promoting the kinetics, as shown in Figure 3. Later, Dan et al. [79] introduced 2–6 nm nickel nanoparticles into MgH_2_. It was shown that the effective improvement in the kinetic performance of MgH_2_ was attributed to the in situ formation of reactive species, such as Mg_2_NiH_0.3_ during the hydrogen absorption and dehydrogenation process. Various reactive substances (e.g., Mg_2_Ni/Mg_2_NiH_4_, Mg_2_NiH_0.3_) formed in situ between metallic nickel and Mg/MgH_2_ during hydrogen absorption and dehydrogenation play an important role in the hydrogen storage performance of Mg/MgH_2_.

On the basis of metallic nickel, scholars have developed nickel-based alloy catalysts. In 2014, Motavalli and Rajabi [80] prepared two different forms of Ni_3_FeMn, a cast alloy and a melt-spun powder, respectively. It was shown that the latter, i.e., melt-spun powder, was harder than the former and had better catalytic effect. Later, El-Eskandarany’s group [65,66,81] successively found that the introduction of Zr_2_Ni and ZrNi_5_ led to a significant enhancement of the kinetics and cycling performance of MgH_2_. At a temperature of 250 °C, MgH_2_-Zr_2_Ni could cycle 2546 hydrogen absorption/dehydrogenation experiments within 1250 h [65]; the MgH_2_-ZrNi_5_ composite system maintained high hydrogen storage performance after 600 cycles within 568 h at 275 °C [66]. In recent years, Ding et al. [67] prepared MgCCo_1.5_Ni_1.5_ by combining various catalytic elements and then introduced it into MgH_2_. It was shown that the alloy material effectively enhanced the kinetic performance of the Mg/MgH_2_ system. Recently, Zhang et al. [68,69] studied a series of Ni-based solid solutions, including Ni-Cu, Ni-Fe and Ni-Co. It was shown that all these Ni-based solid solutions effectively improved the kinetic performance of MgH_2_. Among them, Ni-50%Cu had a better catalytic effect on the dehydrogenation of MgH_2_ than Ni-25%Cu, Ni-75%Cu, Cu and Ni. More importantly, NiCu was uniformly distributed on the surface of MgH_2_, which enhanced the activity of MgH_2_ and also limited the growth of MgH_2_ particles [68]. Moreover, among Ni-25%X (X = Fe, Co, Cu), Ni-25%Co showed the most excellent catalytic effect, and the dehydrogenated MgH_2_ could absorb 5.39 wt.% of hydrogen within 3 min at 275 °C. The excellent hydrogen absorption kinetics can be attributed to the catalytic effect of the in situ formed Mg_2_Ni(Co) [69].

On the basis of metallic nickel, scholars have also developed nickel-based compound catalysts, including NiB [70], NiO [71], NiS [72], Ni_3_S_2_ [77], NiMoO_4_ [73], etc. Liu et al. [70] found that the kinetic performance of 10 wt.% NiB-doped MgH_2_ was greatly improved, because the doping of the catalyst reduced the barrier and the driving force of nucleation. Later, Zhang et al. [71] conducted a series of experiments on the catalysis of nickel-based compounds, including not only NiO as mentioned above, but also Ni_3_C, Ni_3_N, Ni_2_P, etc. It was shown that all these Ni-based catalysts exhibited good catalytic effects, and the dehydrogenation ability of MgH_2_-Ni_3_C was the most remarkable. The introduction of Ni_3_C allowed MgH_2_ to reduce the most dehydrogenation temperature and obtain the highest dehydrogenation amount. Xie et al. [72] found that the dehydrogenation activation energy of MgH_2_ doped with 5 wt.% flower-like NiS particles could be reduced to 64.71 kJ/mol, and the remarkable catalytic performance of NiS can be attributed to the in situ formation of multiphase catalytic substances. Recently, Zeng et al. [77] and Hou et al. [73] reported the catalytic effect of Ni_3_S_2_ and NiMoO_4_ on the hydrogen storage performance of MgH_2_, respectively. Zeng et al. [77] found that Mg_2_Ni and MgS were formed during the first dehydrogenation of Ni_3_S_2_@C with MgH_2_; Hou et al. [73] found that NiMoO_4_ and MgH_2_ produced Mg_2_Ni and Mo after the first dehydrogenation reaction. Therefore, their study showed that the catalytic effect of Ni_3_S_2_ and NiMoO_4_ is equally attributed to the multiple active substances produced during the hydrogen absorption and desorption process.

**Figure 4:**
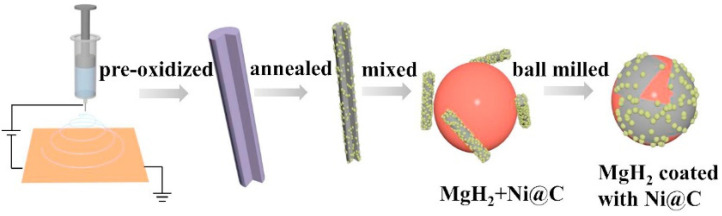
Schematic illustration for the Ni@C formation process and the synergetic catalytic effect on MgH_2_. Copyright 2021, Elsevier. Reproduced with permission from [74].

**Figure 5:**
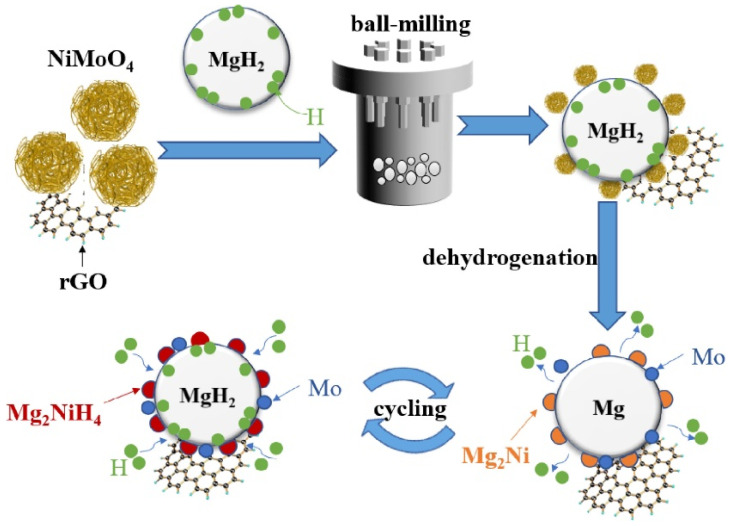
Schematic diagram of the action mechanism of NiMoO_4_ and rGO co-catalyzing MgH_2_ particles. Copyright 2022, Elsevier. Reproduced with permission from [73].

The multiple elements contained in Ni-based alloys and Ni-based compounds usually contribute to the improved kinetic performance of Mg/MgH_2_; however, the defects of Mg/MgH_2_ in cyclic performance gradually emerge. Scholars have found that the use of carbon materials to support nickel-based catalysts not only helps to further improve the kinetic performance of Mg/MgH_2_, but also enhances the stability of Mg/MgH_2_ during cycling. Recently, Meng et al. [74] introduced an electrospinning-based reduction method to generate nickel nanoparticles in situ in carbon nanofibers and investigated the catalytic effect of this composite (Ni@C) on the hydrogen storage performance of MgH_2_. Figure 4 shows the brief synthesis steps of Ni@C. It was shown that the nickel nanoparticles were protected from irreversible fusion and aggregation in subsequent high-temperature pyrolysis because of the presence of carbon nanofibers, leading to the excellent kinetic and cycling performance of MgH_2_. Later, Duan et al. [75] and Hou et al. [76] investigated the catalytic performance of carbon nanotube-supported nickel (CNTs-Ni) and biomass charcoal material-supported nickel (Ni/BC) for MgH_2_, respectively. The study reconfirmed that the introduction of carbon materials was a better choice for improving the hydrogen storage performance of MgH_2_. In fact, Zeng et al. [77] and Hou et al. [73] also focused on the catalytic performance of carbon-supported Ni_3_S_2_ and NiMoO_4_, respectively. Zeng et al. [77] found that the introduction of carbon provided additional catalysis for Mg/MgH_2_ on top of Mg_2_Ni and MgS. To some extent, the catalytic performance of Ni_3_S_2_@C was superior to that of Ni_3_S_2_. Hou et al. [73] showed that the introduction of rGO on top of Mg_2_Ni and Mo effectively inhibited the growth and agglomeration of Mg/MgH_2_ particles during hydrogen absorption and desorption, thus promoting their cyclic stability. Figure 5 shows the synergistic catalytic mechanism of rGO and NiMoO_4_ for Mg/MgH_2_ particles. Carbon-supported nickel-based catalysts not only provide more active catalytic substances to promote the kinetic properties of Mg/MgH_2_, but also enhance the cycling performance of Mg/MgH_2_ by the unique properties of carbon materials.

A series of nickel-based catalysts have been systematically investigated to improve the hydrogen storage performance of Mg/MgH_2_. These Ni-based catalysts all have different degrees of catalytic effects on the hydrogen storage performance of Mg/MgH_2_, and the following points are worth mentioning: (1) The different active substances (e.g., Mg_2_Ni/Mg_2_NiH_4_, etc.) formed between Mg/MgH_2_ and nickel-based catalysts during the hydrogen absorption and desorption cycle have important positive effects on the improvement of the hydrogen storage performance of Mg/MgH_2_. (2) It is difficult to enhance the cycling performance of Mg/MgH_2_ by a single Ni-based catalyst, which can be effectively compensated by the combination of carbon materials with Ni-based catalysts.

### 4.2. Iron (Fe)-Based Catalysts

As early as 1999, iron (Fe) catalysts have been documented to improve the hydrogen storage performance of MgH_2_ [62]. Table 4 summarizes the improvement of the hydrogen storage performance of Mg/MgH_2_ by Fe-based catalysts in the last decade.

In 2013, it was shown that Fe-doped Mg/MgH_2_ had good dehydrogenation/rehydrogenation performance. Mg doped with 5 wt.% Fe could absorb 4.98 wt.% of hydrogen after 15 min of hydrogen absorption at 270 °C, which was second only to the Mg/MgH_2_ system doped with 5 wt.% Ni [63]. Later, Zhang et al. [82] found that iron nanosheets (Fe NS) led to better dehydrogenation/rehydrogenation kinetics of Mg/MgH_2_ compared to iron particles. Recently, Song et al. [93] introduced Fe nanocatalysts into MgH_2_ and found that the operating temperature and activation energy of MgH_2_ was significantly reduced. However, the growth of grains in Mg/MgH_2_-Fe composites during the dehydrogenation/rehydrogenation cycle resulted in capacity loss and kinetic degradation. Nano-Fe can better contribute to the improvement of the kinetic performance of MgH_2_; however, the problem of an unstable cycling performance of MgH_2_ is unavoidable.

Based on metallic iron, scholars have investigated the catalytic performance of iron-based alloys for MgH_2_. Santos et al. [83] found that FeNb alloys had good catalytic performance in the hydrogen absorption and desorption process, while pure metals Fe, Nb and Fe+Nb showed better activity than FeNb alloys in terms of hydrogen absorption and desorption kinetics. FeNb is at a disadvantage because of the low chemical interface energy of the nanointerfaces between MgH_2_/FeNb alloys. It is worth mentioning that the pure metal Fe exhibited significant catalytic performance in this study. In recent years, iron-based alloys, such as Ni_3_Fe [84,91], FeCoNi [85] and CoFeB [86], have been reported, and all these iron-based catalysts exhibited good catalytic performance that can be attributed to the synergistic catalytic effect of multiple active catalytic substances and Fe formed in situ during the hydrogen absorption and desorption process. The presence of multiple catalytic elements together improve the hydrogen absorption and desorption performance of Mg/MgH_2_, but the catalytic performance of the Fe-based alloys still needs to be improved in order to promote the cycle performance of Mg/MgH_2_.

On the basis of metallic iron, scholars have also studied iron-based compound catalysts, such as CoFe_2_O_4_ [94] and FeCl_3_ [87], which showed good catalytic performance. The doping of 7 mol% CoFe_2_O_4_ and 5 wt.% FeCl_3_ led to the reduction of the initial dehydrogenation temperature of MgH_2_ to 260 °C and 290 °C, respectively. Further studies showed that 10 wt.% Fe has a slight advantage over 10 wt.% FeCl_3_ in terms of kinetic catalytic performance under the same conditions of the dehydrogenation process in a short time [87]. Later, Gattia et al. [95] confirmed that the adoption of iron oxides (Fe_2_O_3_ and Fe_3_O_4_), which are less costly and abundant raw materials, as catalysts was also a good choice. Recently, Fu et al. [88] found that MgH_2_-FeNi_2_S_4_ composites had good kinetic performance and the synergistic effect of in situ generated Mg_2_Ni/Mg_2_NiH_4_, MgS and Fe is an important factor to enhance the hydrogen storage performance of Mg/MgH_2_. Recently, Song et al. [89] found that FeOOH was also a good catalyst, which led to a low operating temperature of Mg/MgH_2_ and good hydrogen absorption and desorption kinetics. The MgH_2_-5 wt.% FeOOH composite began to release hydrogen at about 230 °C. In addition, the composite could reversibly absorb 4.4 wt.% hydrogen at 200 °C under a 3.2 MPa hydrogen pressure within 60 min. However, it is difficult for FeOOH to enhance the cycle stability of Mg/MgH_2_, so the catalytic performance of FeOOH needs to be improved. In addition, in this study Song et al. also provided a report on enhancing the catalytic performance of FeOOH, which will be elaborated later. Taken together, the iron-based compound catalysts exhibit good catalytic performance that can be attributed to the active substances in the process of hydrogen absorption and desorption. However, the cycling performance of Mg/MgH_2_ is a difficult point that needs to be improved.

**Figure 6:**
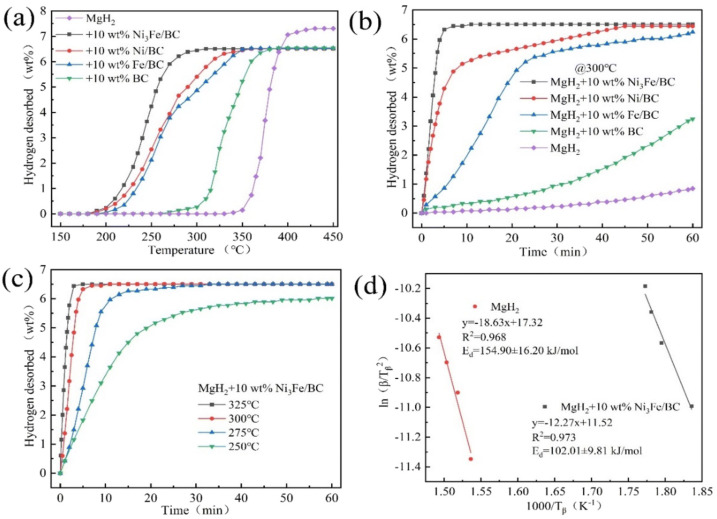
Rising temperature dehydrogenation (**a**) and isothermal dehydrogenation (**b**) curves of pure MgH_2_ and MgH_2_ with 10 wt.% of different catalyst samples, isothermal dehydrogenation curves of MgH_2_ + 10 wt.% Ni_3_Fe/BC composites at different temperatures (**c**), and Kissinger plots of MgH_2_ and MgH_2_ + 10 wt.% Ni_3_Fe/BC (**d**). Copyright 2022, Royal Society of Chemistry. Reproduced with permission from [91].

**Figure 7:**
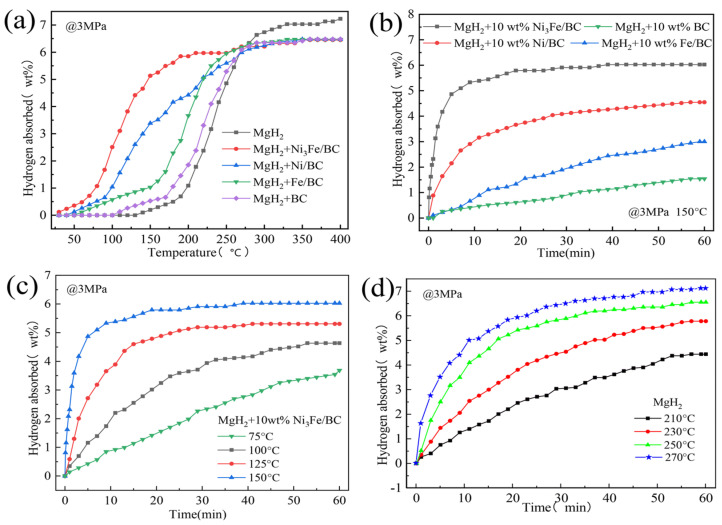
Isothermal hydrogen uptake curves of pure MgH_2_ and MgH_2_ with 10 wt.% of different catalyst samples (**a**), isothermal hydrogen uptake curves of MgH_2_ with 10 wt.% of different catalyst samples under 3 MPa H_2_ pressure at 150 °C (**b**), isothermal hydrogen uptake curves of MgH_2_ + 10 wt.% Ni_3_Fe/BC composite (**c**) and MgH_2_ (**d**) at different temperatures. Copyright 2022, Royal Society of Chemistry. Reproduced with permission from [91].

To improve the cycling performance of Mg/MgH_2_, scholars have adopted carbon-supported iron-based catalysts or used special core-shell structures. Liu et al. [84] investigated the catalytic performance of graphene-supported Ni_3_Fe alloy (Ni_3_Fe/rGO) and comprehensively compared the catalytic performance of Ni_3_Fe/rGO, Ni_3_Fe, Fe/rGO and Ni/rGO. It was shown that the comprehensive catalytic performance of these four catalysts was graded in descending order of Ni_3_Fe/rGO, Ni/rGO, Ni_3_Fe and Fe/rGO. Later, catalysts, such as graphene templated FeCoNi (FeCoNi@GS) [85], carbon nanotubes decorated with CoFeB (CoFeB/CNTs) [86] and Fe–Ni catalyst modified three-dimensional graphene (Fe–Ni@3DG) [90], were developed, and the development of such catalysts demonstrated that the introduction of carbon materials could promote the catalytic effect of iron-based catalysts and enhance the cycling performance of Mg/MgH_2_. Recently, Hou et al. [91] prepared Ni_3_Fe/BC nanocatalysts by the solid-phase reduction method using low-cost biomass carbon (BC) as a carrier and comprehensively compared the catalytic performance of different catalysts, and the catalyst types and performance are shown in Figure 6 and Figure 7. From the two figures, it can be seen that the comprehensive hydrogen storage performance of MgH_2_-10 wt.%Ni_3_Fe/BC is excellent. Notably, the composite could reversibly absorb 5.35 wt.% hydrogen at 150 °C under a 3 MPa hydrogen pressure within 10 min. More importantly, the synergistic catalysis of Mg_2_Ni/Mg_2_NiH_4_ and Fe formed in situ jointly improved the dehydrogenation/rehydrogenation capacity of Mg/MgH_2_. At the same time, iron could also accelerate the mutual conversion of Mg_2_Ni/Mg_2_NiH_4_ to achieve a double promoting effect.

**Figure 8:**
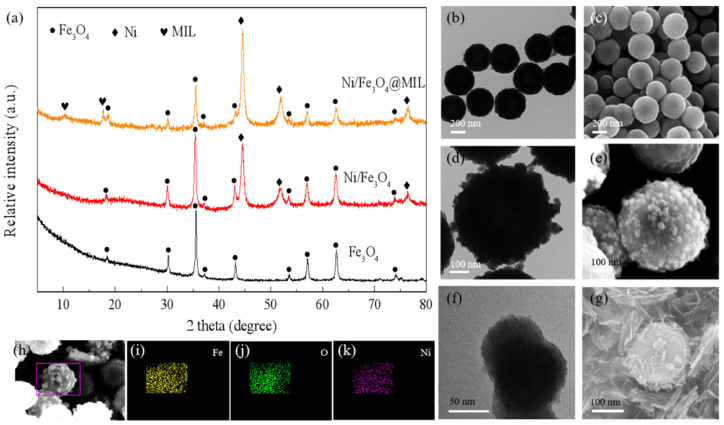
(**a**) XRD patterns of the Fe_3_O_4_, Ni/Fe_3_O_4_ and Ni/Fe_3_O_4_@MIL. TEM and SEM images of Fe_3_O_4_ (**b**,**c**), Ni/Fe_3_O_4_ (**d**,**e**) and Ni/Fe_3_O_4_@MIL (**f**,**g**). EDS mapping of the elemental distribution of (**i**) Fe, (**j**) O and (**k**) Ni corresponding to (**h**) Ni/Fe_3_O_4_. Copyright 2022, Elsevier. Reproduced with permission from [92].

Based on the FeOOH mentioned above, Song et al. [89] also prepared novel graphene-supported FeOOH nanodots (FeOOH NDs@G) and found that MgH_2_-10 wt.% FeOOH NDs@G had excellent hydrogen absorption and desorption kinetics. In addition, the hydrogen capacity of the composite exhibited good cycling stability by maintaining 98.5% of the initial capacity after 20 cycles. The catalytic effect of FeOOH NDs@G could be attributed to the synergistic effect between the graphene nanosheets and the in situ formed Fe. The introduction of carbon materials provides a large number of loading sites for MgH_2_ and catalyst particles, which inhibits their agglomeration and growth, promotes the dissociation and diffusion of hydrogen atoms and ultimately leads to further improvement of MgH_2_ kinetic performance and enhanced cycling performance. In a recent study, Ren et al. [92] prepared a core-shell catalyst, Ni/Fe_3_O_4_@MIL (MIL: metal-organic framework), by wet chemical method, and the morphology and elemental distribution of this structure can be referred to Figure 8. It was shown that this special structure not only provided a suitable reaction site for the catalyst and MgH_2_, but also inhibited the nanoparticle aggregation and the stability of MgH_2_ was maintained, which also provided an important idea for the study of enhancing the hydrogen storage performance of MgH_2_.

A series of iron-based catalysts have been investigated to improve the hydrogen storage performance of Mg/MgH_2_. However, the doping of Mg/MgH_2_ with a single iron-based catalyst does not lead to good cycling performance, while the introduction of carbon materials or the synthesis of special structures effectively enhances the cycling stability of Mg/MgH_2_.

### 4.3. Titanium (Ti)-Based Catalysts

Back in 1999, there was a successful example of titanium (Ti) catalyzing MgH_2_ [62]. Table 5 summarizes the improvement of hydrogen storage performance of Mg/MgH_2_ by titanium-based catalysts in the past decade.

In 2013, it was shown that 5 wt.% Ti led to a hydrogen absorption of 4.3 wt.% for Mg after hydrogenation at 270 °C for 15 min, slightly inferior to Mg/MgH_2_ system doped with the same amount of Ni and Fe catalysts, respectively [63]. Later, both Shahi et al. [96] and Wang et al. [108] found that TiH_2_ was formed when metal Ti was ball-milled with MgH_2_, and the catalytic effect of TiH_2_ promoted the dehydrogenation/rehydrogenation reaction of MgH_2_. In addition, Wang et al. [108] further found that TiH_1.971_ was also formed during co-milling of MgH_2_ with Ti. Active substances, such as TiH_2_ and TiH_1.97_, are important factors for the enhancement of the hydrogen storage performance of MgH_2_. Recently, the above phenomenon was discovered again when Pukazhselvan et al. [109] found that Ti/MgH_2_ generated TiH_2-x_ after intense mechanical grinding, which was converted to TiH_2_ in the subsequent hydrogen absorption and desorption reactions. It is not difficult to see that the titanium has a good catalytic effect, which can be attributed to the active substances TiH_1.971_ and TiH_2_ produced by Ti and MgH_2_ in a series of reactions. 

On the basis of titanium metal, titanium-based alloy catalysts were used to improve the hydrogen storage performance of Mg/MgH_2_. Ren et al. [110] found that TiMn_2_ could effectively improve the hydrogen storage performance of MgH_2_, and no phase change was found in the catalyst during the experiments. Later, catalysts, such as Ti-Mn-Cr [111] and Ti-Cr-Mn-Fe-V [112], were found to produce finer particles after mechanical alloying and, therefore, had greater activity. In addition, the improved dehydrogenation performance of MgH_2_ may be related to a more uniform distribution of alloying elements [111,112]. Recently, Hu et al. [98] found that when MgH_2_−10 wt.%TiMgVNi_3_ was ball-milled in a hydrogen atmosphere, Ti, Mg, V, and Ni formed corresponding hydrides. In further reactions, highly dispersed (Ti,V)H_2_ and in situ formed Mg_2_NiH_4_ nanoparticles were uniformly distributed on the surface of MgH_2_ powder, thus MgH_2_ exhibited excellent kinetic performance. Generally, the polymetallic elements in titanium-based alloys each have a certain catalytic effect, and in the alloying treatment and further reaction, these metal elements can be converted into metals or metal hydrides and other active substances that enhance the hydrogen storage performance of Mg/MgH_2_.

Based on titanium metal, scholars have also developed various titanium-based compound catalysts. Shahi et al. [96] and Wang et al. [108] also explored the catalytic performance of titanium-based compounds. Shahi et al. [96] found that Ti, TiCl_3_, TiO_2_ and TiF_3_ catalysts all improved the Mg/MgH_2_ rehydrogenation kinetics, while the catalytic effect of TiF_3_ was particularly prominent. Nevertheless, the cycling performance of MgH_2_-TiF_3_ composites was deficient, for which the introduction of single-walled carbon nanotubes (SWCNTs) effectively remedied this aspect. Later, the catalytic performance of TiF_3_ was again demonstrated by Wang et al. [108]. Furthermore, by comparing the catalytic performance of TiN, TiO_2_, Ti and TiF_3_, Wang’s team found that the comprehensive performance of these catalysts decreased according to TiF_3_, Ti, TiO_2_ and TiN. More importantly, TiF_3_ and MgH_2_ also formed TiH_1.971_ and TiH_2_ during the co-milling process, and similar phenomena also occurred during the decomposition of MgH_2_-TiO_2_, while TiN’s performance was too stable to produce active substances.

**Figure 9:**
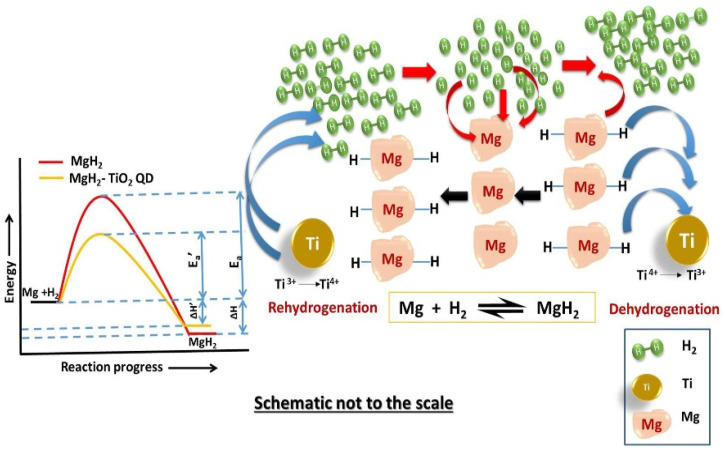
Schematic diagram depicting multiple valencies of Ti in TiO_2_:QDs during dehydrogenation and rehydrogenation of MgH_2_+TiO_2_:QDs sample. Copyright 2021, Elsevier. Reproduced with permission from [99].

The TiH_1.971_ or TiH_2_ generated by some titanium-based compounds with Mg/MgH_2_ in the process of ball milling and hydrogen absorption and desorption provided great help to the improvement of Mg/MgH_2_ hydrogen storage performance. Recently, many scholars have conducted related studies, especially for TiO_2_, to further explore its catalytic performance and mechanism. Pandey et al. [99] found that nanosized titanium dioxide quantum dots (TiO_2_:QDs) had good catalytic effects on the hydrogen absorption and desorption performance and cyclic stability of MgH_2_. In addition, multiple valence states of the Ti were found. As shown in Figure 9, during the dehydrogenation process, Ti^4+^ was reduced to Ti^3+^ and Ti^3+^ was reduced to Ti^2+^, and in this case, the Mg-H bond was unstable and broken, and electrons flowed from Mg-H to Ti. During the rehydrogenation process, Ti^3+^ and Ti^2+^ were converted to Ti^4+^, and these multiple changes of valence states led to a significant increase in the hydrogen absorption performance of Mg/MgH_2_. The dehydrogenated Mg/MgH_2_ system could absorb about 6.10 wt.% hydrogen within 77 s at 280 °C. 

**Figure 10:**
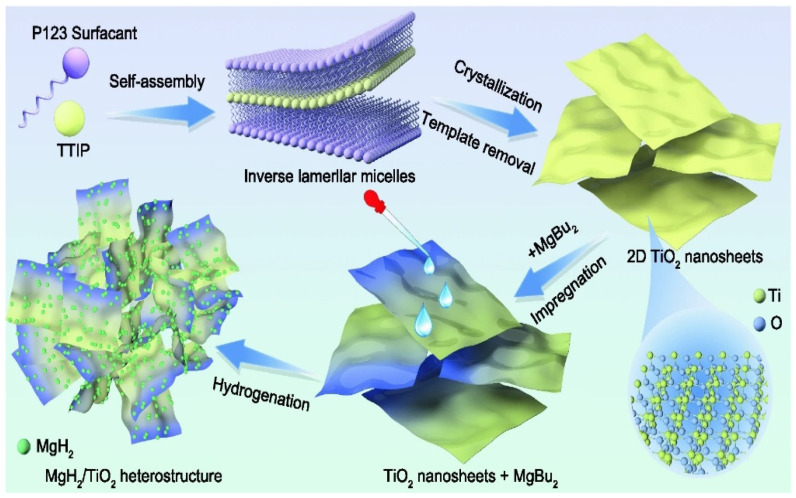
Synthesis process illustration of the MgH_2_/TiO_2_ heterostructure. Copyright 2022, Springer Singapore. Reproduced with permission from [114].

Later, the positive effect of multivalent Ti species in MgH_2_-TiO_2_ on the hydrogen storage performance of MgH_2_ was explored several times. Zou et al. [113] found that the synergistic effect of microwave irradiation and heating contributed to the homogeneous dispersion of defective TiO_2−x_ species around Mg/MgH_2_ and promoted the reduction of Ti^4+^ to lower valence states. TiO_2−x_ and multivalent Ti species are catalysts for electron transfer between Mg^2+^ and H^−^, thus promoting the diffusion of hydrogen. In the same year, Ren et al. [114] explored the hydrogen storage performance of MgH_2_/TiO_2_ heterostructures, and Figure 10 shows the synthesis of two-dimensional TiO_2_ nanosheets (2D TiO_2_ NS), the impregnation of MgBu_2_ and the self-assembly of MgH_2_ nanoparticles on TiO_2_ NS. It was shown that the superior hydrogen storage performance of MgH_2_/TiO_2_ was attributed to a synergistic effect in two aspects. The high specific surface area of 2D TiO_2_ NS provided a channel for the rapid diffusion of hydrogen and inhibited the growth and aggregation of MgH_2_ nanoparticles, thus improving their cyclic stability. On the other hand, the multiphase interface composed of Mg^2+^ and multivalent Ti species provided more diffusion pathways for hydrogen.

It can be seen that the above titanium-based compounds are good catalysts for the challenge of insufficient hydrogen storage performance of Mg/MgH_2_. In general, catalysts that provide more titanium-based active substances can better enhance the hydrogen storage performance of Mg/MgH_2_. TiF_3_, TiO_2_ and other titanium-based compounds can generate titanium hydride, TiO_2-x_ and multivalent titanium species with Mg/MgH_2_, and the synergistic effect of these active substances can enhance the hydrogen storage performance of Mg/MgH_2_.

In the last five years, a range of new titanium-based compound catalysts (MXenes) have been developed [100,101,102,115,116,117,118]. Shen et al. [100] and Zhang et al. [101] developed (Ti_0.5_V_0.5_)_3_C_2_ and TiVO_3.5_, respectively. (Ti_0.5_V_0.5_)_3_C_2_ not only significantly improved the dehydrogenation/rehydrogenation of Mg/MgH_2_, but also effectively enhanced its cycling performance [100]. Later TiVO_3.5_ was synthesized under an oxygen atmosphere at 300 °C by using a solid solution (Ti_0.5_V_0.5_)_3_C_2_ as a precursor. In terms of hydrogen absorption kinetics, TiVO_3.5_ possessed better catalytic performance than (Ti_0.5_V_0.5_)_3_C_2_ [101]. It is worth mentioning that during the ball milling process, both of the above-mentioned catalysts generated the metals Ti and V, thus promoting the dissociation and reorganization of hydrogen molecules.

**Figure 11:**
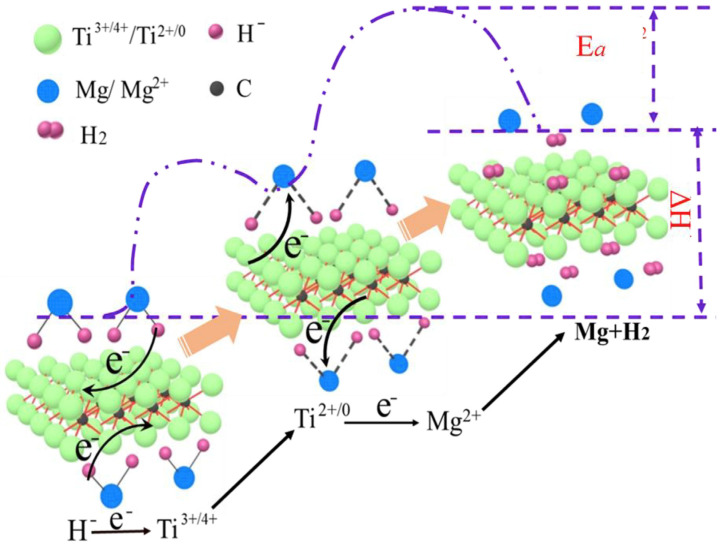
Schematic of the mechanism of Ti_2_C in catalyzing the dehydrogenation of MgH_2_. Copyright 2019, Elsevier. Reproduced with permission from [115].

Since the successive appearance of (Ti_0.5_V_0.5_)_3_C_2_ and TiVO_3.5_, a large number of MXenes catalysts have been reported later, such as Ti_2_C [115], Ti_2_CT_2_ [116], Ti_3_C_2_ [117] and Ti_3_C_2_T_x_ [102,118], etc. The study of Li et al. [115] showed that two-dimensional Ti_2_C had a good catalytic effect on the dehydrogenation process of MgH_2_, as shown in Figure 11, which could be summarized into two points. Ti_2_C MXene itself had good hydrogen adsorption ability and thermal conductivity. On the other hand, the surface Ti atoms with multivalence served as the intermediate for electrons shifting between H^−^ and Mg^2+^. Recently, the catalytic effect of Ti_2_C on MgH_2_ was demonstrated again. Huang et al. [116] found that this catalytic effect was not only due to the Ti atoms serving as an intermediary for electron transfer between Mg^2+^ and H^−^, but also due to the catalytic effect of TiH_2_ formed in situ at the Ti_2_C/MgH_2_ interface, which together promoted the dehydrogenation reaction of MgH_2_. In addition, Ti_2_C could enhance the dehydrogenation reaction of MgH_2_ more effectively than Ti_2_CT_2_ (T = O, F, OH). The different surface functional groups in Ti_2_CT_2_ also had a significant effect on the dehydrogenation performance of MgH_2_, and Ti_2_C(OH)_2_ had a better catalytic performance than Ti_2_CF_2_. 

Recently, Wu et al. [117] synthesized a composite hydrogen storage system of MgH_2_ with multilayer Ti_3_C_2_ (ML-Ti_3_C_2_). It was shown that the electron transfer generated by Ti and multivalent Ti in this hydrogen storage system promoted the dissociation or recombination of hydrogen molecules. Later, Gao et al. [102] prepared Ti_3_C_2_T_x_ catalysts in a similar way to the previous (Ti_0.5_V_0.5_)_3_C_2_ [100]. The accordion-like Ti_3_C_2_T_x_ (F-Ti_3_C_2_T_x_) was obtained by removing the Al layer in Ti_3_AlC_2_ first, and then the paper-like Ti_3_C_2_T_x_ (E-F-Ti_3_C_2_T_x_) was obtained by ultrasonic stripping and filtration. Finally, the two different morphologies of Ti_3_C_2_T_x_ were introduced into MgH_2_ separately. Among them, F-Ti_3_C_2_T_x_ has more edge surfaces in contact with MgH_2_, and E-F-Ti_3_C_2_T_x_ has more base surfaces in contact with MgH_2_. It was shown that different exposure surfaces were the dominant factors affecting the catalytic activity of Ti_3_C_2_T_x_, and F-Ti_3_C_2_T_x_ with more exposed edge surfaces showed better catalytic activity promoting more in situ formation of metallic Ti and thus better MgH_2_ kinetics. In the same year, Gao’s group [118] again reported the catalytic performance of Ti_3_C_2_T_x_, which was the first report of Ti_3_C_2_T_x_ with different residual Al. It was shown that the residual Al in Ti_3_C_2_T_x_ contributed to its catalytic activity. The unique Ti-Al metal bond could change the electronic structure of Al, which contributed to the desorption and absorption of H atoms. Invariably, multivalent Ti can further enhance the kinetic performance of MgH_2_.

Among the many titanium-based catalysts, titanium-based MXenes have been widely used to improve the hydrogen storage performance of Mg/MgH_2_ only in recent years. Titanium-based MXenes have good hydrogen storage capacity and thermal conductivity by themselves. On the other hand, the in situ generation of various active substances is an important factor to promote the dissociation or recombination of hydrogen molecules. Among them, Ti and multivalent Ti are in a very special position, as they are the intermediaries of electron transfer between H^−^ and Mg^2+^, which are the key factors to enhance the hydrogen storage performance of Mg/MgH_2_. Therefore, making the in situ formation of more metallic Ti and multivalent Ti species in titanium-based MXenes is the focus of promoting the performance enhancement of Mg/MgH_2_.

As mentioned previously, carbon nanotube-supported titanium-based catalyst [96] enhanced the kinetic and cycling performance of MgH_2_. In recent years, carbon- supported titanium-based catalysts have maintained a high degree of heat, including not only carbon-supported alloys, but also carbon-supported compounds, and the carbon materials used include carbon nanotubes (CNTs), graphene (Gr), and so on. Zr_0.4_Ti_0.6_Co-CNTs [119], TiFe/CNTs [97] and Ti-Ni-Fe@Gr [103] have excellent catalytic performance: on top of the multi-element co-catalysis of MgH_2_ by the alloy, the introduction of carbon materials can inhibit the aggregation of nanoparticles, which further enhances not only the kinetics but also the cycling performance. In addition, scholars used different types of carbon materials to support titanium-based compounds, and by this method, catalysts, such as TiH_2_@Gr [104], TiO_2_@Gr [104], TiO_2_@C [105] and TiO_2_@rGO [120], were prepared. Among them, Verma et al. [104] found that TiH_2_@Gr was more effective than Ti@Gr and TiO_2_@Gr for the catalysis of MgH_2_. The excellent catalytic performance of these carbon-supported titanium-based compound catalysts is mainly due to the addition of carbon materials as co-catalysts based on the catalysis of titanium-based active substances, such as polyvalent titanium, which inhibits the aggregation of nanoparticles, thereby further enhancing the kinetic performance of MgH_2_ and also its cycling performance.

**Figure 12:**
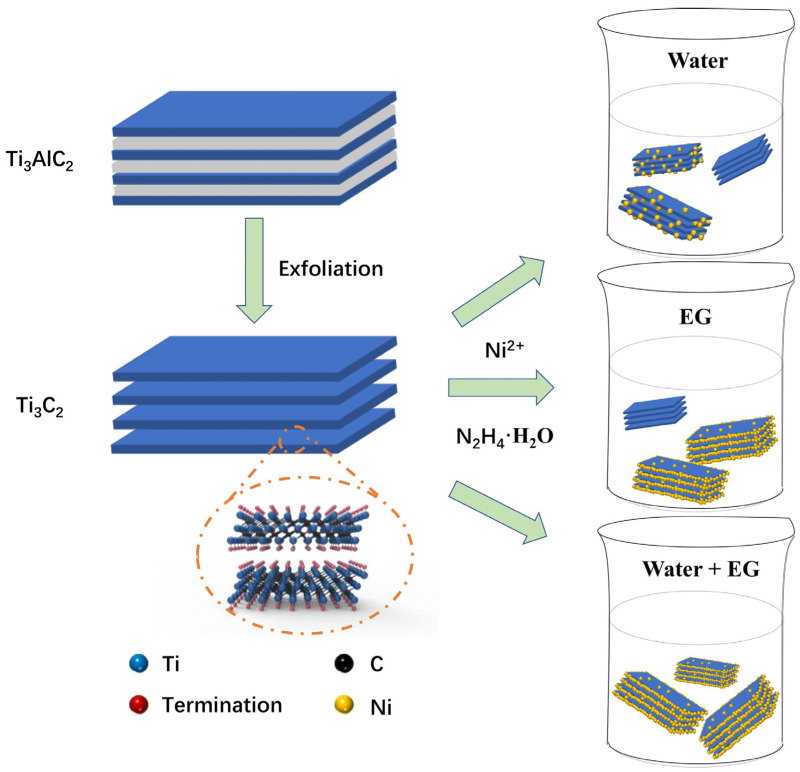
The synthesis procedures of sandwich-like Ni/Ti_3_C_2_ catalysts. Copyright 2021, Elsevier. Reproduced with permission from [106].

Recently, Ti-based MXenes, not only as catalysts but also as carriers of various active catalysts (similar to the loading role of carbon materials), have been focused on by scholars with most studies on Ti_3_C_2_ and Ti_3_C_2_T_x_. Gao et al. [106] first removed Al from Ti_3_AlC_2_ to obtain Ti_3_C_2_ and then reduced Ni nanoparticles to the surface of Ti_3_C_2_ matrix by chemical reduction, thereby preparing a sandwich-like Ni/Ti_3_C_2_ catalyst, and its synthesis process and morphology are shown in Figure 12. It was shown that the Ni nanoparticles with the smallest size and the best dispersion on the surface of Ti_3_C_2_ substrate had the best catalytic activity, and the electronic interactions from the rich interface between Ni and Ti_3_C_2_ could greatly improve the hydrogen storage performance of MgH_2_. In addition, the electron transfer of multivalent Ti and the unique structure of Ni/Ti_3_C_2_ were also important factors for the catalytic performance. In the same year, Gao’s group [107] self-assembled TiO_2_ nanoparticles (M-TiO_2_) on several layers of Ti_3_C_2_T_x_ (FL-Ti_3_C_2_T_x_) by a one-step sonication method, which could alleviate the heavy accumulation of FL-Ti_3_C_2_T_x_ and the agglomeration of M-TiO_2_ nanoparticles, resulting in a large number of interfaces between them. The abundant interface not only serves as a hydrogen diffusion channel, but also the electron transfer at the interface can enhance the catalytic activity of the whole heterogeneous structure. In addition, multivalent Ti can effectively enhance the reversible hydrogen storage performance of MgH_2_. Recently, Ti_3_C_2_ and FL-Ti_3_C_2_T_x_ have also been used to load catalytic substances, such as PrF_3_ nanoparticles [121] and Ni@C nanosheets [122], respectively. PrF_3_/Ti_3_C_2_ prepared by Wang et al. [121] exhibited excellent catalytic activity for hydrogen storage of MgH_2_, not only because of the significant enhancement of Ti_3_C_2_ MXene by PrF_3_, but more so because of the facilitation effect of multivalent Ti-species. The Ni@C/FL-Ti_3_C_2_T_x_ prepared by Peng et al. [122] had efficient catalytic performance, not only due to the catalytic performance of FL-Ti_3_C_2_T_x_ itself, but also because of the active substances (Mg_2_NiH_4_ and small-size, highly dispersible Ti nanoparticles) formed in situ during the reaction of MgH_2_-Ni@C/FL-Ti_3_C_2_T_x_.

Titanium-based MXenes, as carriers of active substances, share similarities with carbon materials both acting as carriers and co-catalysts to improve the kinetic and cycling performance of Mg/MgH_2_. However, the difference with carbon materials is that instead of carbon, titanium-based MXenes provide highly dispersed Ti and multivalent Ti species during the reaction with Mg/MgH_2_.

The catalytic performance of titanium-based catalysts has been systematically studied by scholars, and the following points are worth mentioning in the comprehensive development history and trends of titanium-based catalysts: (1) Titanium metal, some titanium-based compounds (TiF_3_, TiO_2_, etc.) and titanium-based alloys generate a variety of titanium-based active substances (TiO_2−x_, TiH_1.971_ and multivalent titanium species, etc.) or active substances formed by other elements in the reaction with Mg/MgH_2_ have good catalytic effects on Mg/MgH_2_. Therefore, the introduction of these titanium-based catalysts, which are easy to generate active substances, is more helpful to enhance the kinetic performance of Mg/MgH_2_. (2) Carbon-supported titanium-based alloys or carbon-supported titanium-based compounds have further catalytic performance. Based on the original catalyst, the carbon material acts as a co-catalyst, which not only enhances the kinetic performance of Mg/MgH_2_, but also strengthens its cycling performance. (3) Titanium-based MXenes can be used to significantly improve the hydrogen storage performance of Mg/MgH_2_, mainly because of two aspects: as catalysts and as carriers of catalytic substances. As catalysts, the in situ generation of a variety of active substances (Ti and multivalent Ti, etc.) from titanium-based MXenes is an important factor in enhancing the kinetics. As catalysts, similar to carbon materials, they can be used to load active substances and act as co-catalysts to improve the kinetics and cycling performance of Mg/MgH_2_. Unlike carbon materials, titanium-based MXenes provide highly dispersed Ti as well as multivalent titanium during the reaction with Mg/MgH_2_. Therefore, titanium-based MXenes, which will be one of the main titanium-based catalysts to be studied in the future, have good prospects. 

### 4.4. Vanadium(V)-Based Catalysts

In 1999, the study of vanadium (V) catalyzing MgH_2_ has been reported [61]. Overall, vanadium possesses a good catalytic effect and can rank well among many metal catalysts [62]. Later, vanadium-based catalysts were gradually investigated, and Table 6 summarizes the improvement of the hydrogen storage performance of Mg/MgH_2_ by vanadium-based catalysts in the past decade.

In the last decade, da Conceição et al. [123] found that V, VC and VCl_3_ all showed good catalytic effects, among which, VC and VCl_3_ better enhanced the kinetic performance of MgH_2_. It is worth mentioning that MgH_2_ doped with just 5 wt.% VCl_3_ exhibited rapid kinetics and good hydrogen capacity. The Mg/MgH_2_-5 wt.% VCl_3_ system could absorb about 5.4 wt.% hydrogen within 2.5 min at 300 °C. Thus, VCl_3_ has a significant catalytic effect and can reduce the use of pure V to reduce the cost. Later, Milošević et al. [124] explored the hydrogen storage performance of MgH_2_-VO_2_(B) and found that during hydrogen absorption and desorption, part of VO_2_(B) was reduced to V at high temperatures and a VH_2_ phase appeared. The vanadium metal together with multivalent vanadium (VO_2_/VH_2_ system) contributed to the kinetic performance of MgH_2_. Later, many multi-element vanadium-based catalysts were developed, such as the alloy V_45_Zr_20_Ni_20_Cu_10_Al_3_Pd_2_ [125], compounds VB_2_ [126], V_4_Nb_18_O_55_ [127] and Ni_3_(VO_4_)_2_ [129].

**Figure 13:**
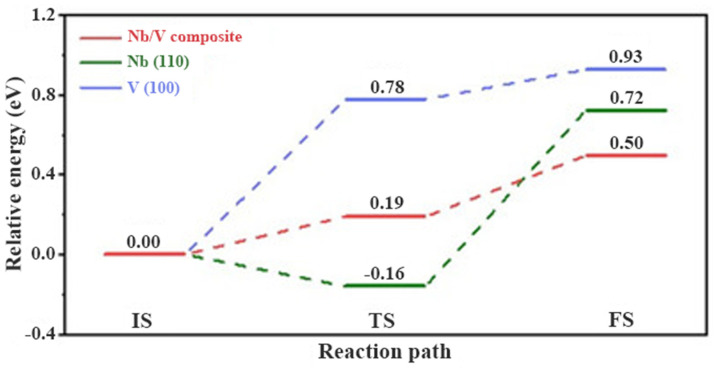
Calculated energy profiles for the H_2_ desorption of MgH_2_ on V (100), Nb (110) and Nb/V composites, respectively. Copyright 2022, Wiley Online Library. Reproduced with permission from [127].

El-Eskandarany et al. [125] investigated the catalytic performance of two different forms of V_45_Zr_20_Ni_20_Cu_10_Al_3_Pd_2_ (intermetallic compound powders and metallic glassy powders) for MgH_2_. It was shown that the latter, compared to the former, exhibited better catalytic performance for the kinetics of the Mg/MgH_2_. MgH_2_ doped with 10 wt.% metallic glassy V_45_Zr_20_Ni_20_Cu_10_Al_3_Pd_2_ could desorb hydrogen about 5.5 wt.% hydrogen within 3 min at 180 °C. Recently, Zang et al. [129] synthesized Ni_3_(VO_4_)_2_ and introduced it into MgH_2_. It was shown that the hydrogen storage performance of MgH_2_ performed well based on the dual catalysis of Mg_2_Ni and V generated by the reaction. In addition, this study identified the intermediate active species NiV_2_O_4_ for the first time. Later, Pang et al. [126] found that VB_2_ nanoparticles with dual catalytic function could significantly enhance the kinetics of MgH_2_. It was shown that during ball milling and dehydrogenation, VB_2_ reacted with MgH_2_ to form V and MgB_2_ in situ. V acted as the active species, providing the nucleation site and reducing the apparent activation energy, while MgB_2_ had some hydrogen absorption capacity. Therefore, the synergistic effect of V and MgB_2_ is an important factor for VB_2_ to improve the hydrogen storage performance of MgH_2_. In a recent study, Meng et al. [127] prepared V_4_Nb_18_O_55_ microspheres as a modified catalyst for MgH_2_. It was shown that the synergistic effect of V^5+^ and Nb^5+^ was clear, and V_4_Nb_18_O_55_ composed of V^5+^ and Nb^5+^ had a better catalytic effect than V_2_O_5_ and Nb_2_O_5_. More importantly, the homogeneous construction of the Nb/V interface not only preserved the ability of Nb to weaken the Mg-H bond, but also alleviated the strong adsorption ability of metal Nb on hydrogen atoms resulting in a relative energy barrier of only 0.5 eV for the whole dehydrogenation process of MgH_2_, which was 0.22 and 0.43 eV lower than that of Nb and V, respectively, as shown in Figure 13. Therefore, V_4_Nb_18_O_55_ effectively plays the advantages of V and Nb elements and significantly improves the hydrogen storage performance of MgH_2_.

The catalytic effect of multiple elements in the above vanadium-based catalysts effectively improves the hydrogen storage performance of Mg/MgH_2_, which is better reflected by the carbon-supported vanadium-based catalysts, mainly due to the synergistic effect of the V-based active elements with carbon. Wang et al. [128] synthesized nano-V_2_O_3_@C, in which V_2_O_3_ nanoparticles were loaded on cubic carbon nanoboxes. It was shown that the doping of 9 wt.% nano-V_2_O_3_@C resulted in a significant decrease in the operating temperature and a significant improvement in the kinetic performance of MgH_2_. It is worth mentioning that V_2_O_3_ was reduced to metal V during ball milling and initial heating, and V remained unchanged during hydrogen absorption and desorption, thus promoting the breakage of Mg-H bonds and improvement of kinetic performance.

Many vanadium-based catalysts have been used to improve the hydrogen storage performance of Mg/MgH_2_; the following points are worth mentioning: (1) Considering the high cost of pure V, vanadium-based compounds or alloys with better catalytic performance can be adopted to reduce the cost. (2) Multi-element vanadium-based alloys or compounds generally have better catalytic performance due to the synergistic catalytic performance of in situ generated V metal, multivalent V and other active substances that can complement each other and together contribute to the kinetic performance of Mg/MgH_2_. (3) Carbon-supported vanadium-based catalysts can achieve better multi-catalytic effects due to the synergistic effect of vanadium-based active elements with carbon. However, the combination of vanadium-based catalysts with carbon materials has been relatively little studied, and it would be good to try to combine more vanadium-based catalysts with carrier materials, such as carbon materials, in the future development of vanadium-based catalysts.

### 4.5. Manganese (Mn)-Based Catalyst

In the late 1990s, Liang’s group [62] conducted research on a Mn catalyst. Later, although manganese-based catalysts were studied, they were still relatively rare compared to Ti, Fe and Ni. Table 7 summarizes the improvement of hydrogen storage performance of Mg/MgH_2_ by manganese-based catalysts in recent years.

In recent years, Sun et al. [130] found that doping of 10 wt.% submicron-Mn could achieve good catalytic effect. At this time, MgH_2_ was rapidly dehydrogenated by 6.6 wt.% within 8 min at 300 °C. At a temperature of 100 °C and a hydrogen pressure of 3 MPa, the fully dehydrogenated MgH_2_ reabsorbed nearly 3.0 wt.% of hydrogen within 30 min. More importantly, the submicron-Mn-doped MgH_2_ exhibited good cyclic stability. Recently, Chen et al. [131] found that the initial dehydrogenation temperature of MgH_2_ was reduced from 355 °C to 175 °C after the introduction of 10 wt.% nano-Mn, which was better than that of 10 wt.% submicron-Mn. Moreover, the dehydrogenated material could absorb hydrogen even at a low temperature of 50 °C. It is worth mentioning that in addition to the kinetic performance, the cyclic performance of MgH_2_ is also improved.

Based on manganese metal, scholars have developed some manganese-based alloy catalysts. Meena et al. [137] found that MgH_2_ with the introduction of NiMn_9.3_Al_4.0_Co_14.1_Fe_3.6_ obtained a lower working temperature. Later, Zhang et al. [132] used LaNi_4.5_Mn_0.5_ to improve the hydrogen storage performance of MgH_2_ and found that LaNi_4.5_Mn_0.5_ submicro-particles had a significant catalytic effect. It was shown that this catalyst could enable MgH_2_ to start dehydrogenation at a lower temperature (175 °C) and enhance its kinetic performance. At 300 °C, MgH_2_ doped with 10 wt.% submicronLaNi_4.5_Mn_0.5_ could desorb 6.6 wt% H_2_ within 6 min. Moreover, the fully dehydrogenated system could absorb 4.1 wt.% H_2_ within 10 min at 150 °C. It is worth mentioning that the synergistic effect between the in situ formed Mg_2_Ni/Mg_2_NiH_4_, Mn and LaH_3_ is the key to enhance the hydrogen storage performance.

Based on manganese metal, scholars have also explored the catalytic performance of manganese-based compounds. Sun’s team [130] found that MnCl_2_ could reduce the initial dehydrogenation temperature of MgH_2_ from 315 °C to 225 °C. Later, Zhang et al. [133] found good kinetic performance of MgH_2_ with the addition of 10 wt.% Mn_3_O_4_ nanoparticles. In addition, the cycling stability of MgH_2_-Mn_3_O_4_ was good and its hydrogen storage performance could be well maintained in 20 cycles. More importantly, Mn_3_O_4_ with MgH_2_ was reduced to metal Mn in the reaction, Mg_0.9_Mn_0.1_O was also generated along with it and the Mg-H bond was subsequently weakened. Therefore, active substances, such as Mn and Mg_0.9_Mn_0.1_O are the key for Mn_3_O_4_ to improve the hydrogen storage performance of MgH_2_. Recently, MnO achieved a catalytic effect similar to that of Mn_3_O_4_. Fu et al. [134] found that MgH_2_-MnO generated Mn and Mg_0.9_Mn_0.1_O after the first dehydrogenation, which promoted the breakage of the Mg-H bond and improved the reversible hydrogen storage performance of MgH_2_. Wang et al. [135] found that MnS could significantly improve the hydrogen absorption and desorption kinetics of Mg/MgH_2_, and its catalytic effect could be attributed to the in-situ formation of the active substance metal Mn. The catalytic performance of MnMoO_4_ was demonstrated in a recent study in which Zhang et al. [136] showed that MnMoO_4_ rod catalysts could effectively improve the hydrogen storage performance of MgH_2_. In this study, MnMoO_4_ reduced the initial dehydrogenation temperature of MgH_2_ and enhanced the kinetic and cyclic performance of MgH_2_. It is worth mentioning that the synergistic effect of the in situ generated Mn and MgMo_2_O_7_ is the key to improve the hydrogen storage performance of MgH_2_.

In fact, Fu’s team [134] synthesized carbon-supported MnO nanocomposites (MnO@C) on the basis of MnO. It was shown that MgH_2_-10 wt.%MnO@C had superior reversible hydrogen storage performance compared to MgH_2_ and MgH_2_-10 wt.%MnO due to the co-catalytic effect of Mn, Mg_0.9_Mn_0.1_O and C during the reaction. 

Combining the above manganese-based catalysts, the following points can be derived: (1) Multi-element manganese-based alloys or compounds have good catalytic performance because of the active materials formed by different elements in the process of hydrogen absorption and desorption (such as Mn, Mg_0.9_Mn_0.1_O and LaH_3_, etc.), and their synergistic effect is the key to enhance the hydrogen storage performance of Mg/MgH_2_. (2) Carbon materials have a certain gain for Mn-based catalysts, and the reversible hydrogen storage performance of Mg/MgH_2_ can be further improved under the catalytic effect of carbon materials. However, the study of carbon-supported Mn-based catalysts is still relatively small and inconvenient to draw systematic conclusions, which can be extended in this direction to expand more possibilities of Mn-based catalysts.

### 4.6. Summary of Catalytic Approach

Facing the challenge of insufficient kinetic performance and cycling performance of Mg/MgH_2_, scholars have studied a large number of metal-based catalysts, including nickel-based, iron-based, titanium-based, vanadium-based and manganese-based catalysts, which have all enhanced the kinetic performance and cycling performance of Mg/MgH_2_ to different degrees. The following points can be derived from the above studies.

(1) Catalyst particle size, doping amount and ball milling time are important factors to improve the hydrogen storage performance of Mg/MgH_2_; however, catalyst materials with small particle size, high doping amount and long ball milling time should not be pursued blindly, and the pros and cons need to be weighed otherwise it may be counterproductive.
(2) For the enhancement of the hydrogen storage performance of Mg/MgH_2_, the active substances (Mg_2_Ni/Mg_2_NiH_4_, Fe, multivalent titanium, V, Mg_0.9_Mn_0.1_O, etc.) generated in the reaction between metal-based catalysts and Mg/MgH_2_ have an important positive impact. Usually, multi-element catalysts have better catalytic effects than single metal catalysts because the synergy between multiple active substances formed by multiple elements can complement each other and together enhance the kinetic performance of Mg/MgH_2_.
(3) A single metal-based catalyst is certainly excellent, but it is difficult to enhance the cycling performance of Mg/MgH_2_, while the combination of carbon material and catalyst can effectively limit the particle size and inhibit its growth and agglomeration, thus further enhancing the kinetic and cycling performance of Mg/MgH_2_. Here, titanium-based MXenes have to be mentioned, which can be used not only as catalysts to improve the kinetic performance of Mg/MgH_2_, but also as carriers of catalytic substances to improve the kinetic and cyclic performance of Mg/MgH_2_. Regardless of the use, the catalytic effect of titanium-based MXenes on Mg/MgH_2_ comes mainly from the in situ generation of a variety of active substances, such as metal Ti and multivalent Ti species. Therefore, titanium-based MXenes are of great significance for the improvement of hydrogen storage performance of Mg/MgH_2_, which will be one of the main research titanium-based catalysts and materials for supporting active substances in the future.

## 5. Conclusions and Prospects

For the sake of sustainable development and the development of hydrogen energy, scholars have been working hard to explore and obtain high-efficiency hydrogen storage materials. Among a large number of hydrogen storage materials, Mg/MgH_2_ stands out because of its unique advantages of high reversible hydrogen storage capacity, high reliability and high exploitability. However, Mg/MgH_2_ has problems, such as over-stable thermodynamic performance, high temperature of hydrogen desorption and slow kinetic performance. Research on the modification of Mg/MgH_2_ is advancing at high speed and high quality.

Enhancing the comprehensive hydrogen storage performance of Mg/MgH_2_ is a long and arduous challenge, and the success of modification measures, such as alloying, nanosizing and catalyst doping, has added much hope to this challenge. Scholars have been able to effectively improve the thermodynamic performance of Mg/MgH_2_ through alloying and nanosizing, while catalyst doping can largely enhance its kinetic and cyclic performance. In the future, catalyst doping is likely to remain the main improvement technique and alloying and nanosizing can assist it to better improve the hydrogen storage performance of Mg/MgH_2_. Based on the current hot research, the following points are worth mentioning: (1) In order to ensure that the catalysts can fully play their roles, the morphology, size and other indicators of the catalysts are very important. Based on this, nanoscale catalysts would be a better choice. (2) The active material generated during the reaction between the catalyst and Mg/MgH_2_ is very important; therefore, a multi-element catalyst that can generate more active material would be a better choice. (3) A single catalyst, which does not necessarily improve the cycling performance of Mg/MgH_2_, can be assisted by the introduction of carrier materials such as carbon materials to enhance the cycling stability of Mg/MgH_2_. Based on this, titanium-based MXenes would be an additional good choice, which is not only an excellent catalyst but also a good carrier material. Moreover, if more carrier-based materials can be developed and active substances with excellent catalytic ability can be combined with them, it will help the advancement of research in Mg/MgH_2_ and even the whole field of hydrogen storage. (4) Last but not least, in order to systematically enhance the hydrogen storage performance of Mg/MgH_2_ in future studies, on the basis of alloying or nanosizing to improve the thermodynamic performance of Mg/MgH_2_, catalysts with superior performance can be adopted to substantially enhance the kinetic and cycling performance of Mg/MgH_2_.

This paper mainly discusses the research progress and trends of hydrogen storage performance of Mg/MgH_2_ in the past decade and its improvement measures, hoping to provide ideas and help for future research on Mg/MgH_2_ and even many hydrogen storage materials. It is believed that with continued efforts in the right research direction and a good combination of the advantages of Mg/MgH_2_ and improvement measures, the production of Mg/MgH_2_ with superior hydrogen storage performance will not be far away.

## Figures and Tables

**Table 1:** Improvement of hydrogen storage performance of Mg/MgH_2_ by alloying treatment.

System	Hydrogen Absorption Conditions	Hydrogen Absorption Capacity (wt.%)	Dehydrogenation Conditions	Dehydrogenation Capacity (wt.%)	Dehydrogenation Ea (kJ/mol)	Ref.	Year
Mg/MgH_2_	>300 °C/>3 MPa	~7.6	>300 °C	~7.6	160	[35]	2021
0.75Mg-0.25Ti	270–375 °C	~4.2	270–375 °C	~4.2	53.6	[39]	2018
0.75Mg-0.25V	300–360 °C	~4	300–360 °C	~4	96.3	[39]	2018
0.75Mg-0.25Nb	300–350 °C	~3.8	300–350 °C	~3.8	141.3	[39]	2018
Mg_2_Ni	375 °C/5 min	3.44	——	——	209.65	[40]	2018
Mg-Al	260–375 °C	5.8	——	——	164–169	[41]	2022
Mg_90_Ce_3_Ni_7_	100 °C/30 min	>3.5	280 °C/10 min	>5	72.2	[42]	2022
Pr-Mg-Ni	100 °C/60 min	3.38	280 °C/13.7 min	~4.0	——	[43]	2022
Gd_5_Mg_80_Ni_15_	100 °C/40 min	>3	280 °C/10 min	>4.8	75.07	[44]	2022
Mg-Y-Zn	320 °C/60 min	>5.0	360 °C/23 min	6.31	——	[45]	2022
Mg-Ni-Y	300 °C/60 min	6	360 °C/5 min	6	91.8	[46]	2022

**Table 2:** Improvement of hydrogen storage performance of Mg/MgH_2_ by nanosizing.

Preparation Methods	Particle Size (nm)	Hydrogen Absorption Conditions	Hydrogen Absorption Capacity (wt.%)	Dehydrogenati on Conditions	Dehydrogenation Capacity (wt.%)	Dehydrogenation Ea (kJ/mol)	Ref.	Year
Ball milling	500–600	——	——	——	——	——	[51]	2006
Gas-phase reaction	~40	4 MPa/287 °C/10 min	>5	377 °C/10 min	5	114	[52]	2011
Chemical reduction	38	300 °C/7 min	~6.2	——	——	160	[53]	2011
Chemical reduction	32	300 °C/2.3 min	~6.2	——	——	131	[53]	2011
Chemical reduction	25	300 °C/1 min	~6.2	——	——	126	[53]	2011
MgH_2_/CAs	6–20	1.8 MPa/300 °C/15 min	~1.5	——	——	——	[54]	2014
MgH_2_@BCNTs	15–20	8 MPa/250 °C/5 min	5.79	275 °C/60 min	5.70	97.94	[55]	2019
MgH_2_@CCNTs	15–20	8 MPa/250 °C/15 min	5.79	275 °C/60 min	3.18	——	[55]	2019
MgH_2_@CSC	23	2 MPa/250 °C/5 min	~5.0	325 °C/10 min	5.4	120.19	[56]	2020

**Table 3:** Hydrogen storage performance of Mg/MgH_2_ system doped with nickel-based catalysts.

Catalysts	Hydrogen Absorption Conditions	Hydrogen Absorption Capacity (wt.%)	Dehydrogenation Conditions	Dehydrogenation Capacity (wt.%)	Dehydrogenation Ea (kJ/mol)	Ref.	Year
5 wt.%Ni	1.5 MPa/270 °C/15 min	5.0	340 °C	——	72.81	[63]	2013
5 wt.%Ni	3 MPa/125 °C/20 min	4.6	300 °C/3 min	6.7	83.9	[64]	2021
10 wt.%Zr_2_Ni	250 °C/1.9 min	5.1	250 °C/10.2 min	5.9	——	[65]	2015
10 wt.%ZrNi_5_	275 °C/1 min	5.3	275 °C/10 min	5.2	110.06	[66]	2017
MgCCo_1.5_Ni_1.5_	3 MPa/150 °C/60 min	5.5	325 °C/10 min	5	39.6	[67]	2020
Ni-50%Cu	3 MPa/250 °C/30 min	4.37	300 °C/15 min	5.14	——	[68]	2020
Ni-25%Cu	250 °C/3 min	3.73	300 °C/10 min	4.42	——	[69]	2022
10 wt.%NiB	——	——	300 °C/10 min	6.0	59.7	[70]	2012
5 wt.%Ni_3_C	——	——	300 °C/20 min	6.2	97.8	[71]	2017
5 wt.%NiS	150 °C/10 min	3.5	300 °C/10 min	3.1	64.71	[72]	2017
10 wt.%NiMoO_4_	3 MPa/125 °C/10 min	4.4	300 °C/3 min	6.0	119.47	[73]	2022
10 wt.%Ni@C	300 °C/1.3 min	4.78	300 °C/10 min	4.8	93.08	[74]	2021
CNTs-Ni	6 MPa/200 °C/30 min	7.2	300 °C/15 min	7.29	74.8	[75]	2022
10 wt.%Ni/BC-3	3 MPa/125 °C/60 min	5	300 °C/3.5 min	6.04	72.41	[76]	2022
Ni_3_S_2_@C-4	150 °C/10 min	6.08	300 °C/8 min	6	115.2	[77]	2021
10 wt.%NiMoO_4_/rGO	3 MPa/125 °C/10 min	4.2	300 °C/3 min	6.0	——	[73]	2022

**Table 4:** Hydrogen storage performance of Mg/MgH_2_ system doped with iron-based catalysts.

Catalysts	Hydrogen Absorption Conditions	Hydrogen Absorption Capacity (wt.%)	Dehydrogenation Conditions	Dehydrogenation Capacity (wt.%)	Dehydrogenation Ea (kJ/mol)	Ref.	Year
5 wt.%Fe	1.52 MPa/270 °C/15 min	4.98	310 °C	——	60.88	[63]	2013
5 wt.%FeNS	3.2 MPa/300 °C/0.5 min	5.87	300 °C/10 min	5.44	40.7	[82]	2019
7 wt.%FeNb granule	2 MPa/350 °C/2 min	5.5	350 °C/2 min	1.2	——	[83]	2014
5 wt.%Ni_3_Fe	3 MPa/100 °C/8.3 min	2.2	250 °C/20 min	3.4	82.1	[84]	2020
5 wt.%FeCoNi	1.5 MPa/290 °C/1.65 min	4.40	290 °C/8.5 min	4.47	90.24	[85]	2020
10 wt.%CoFeB	5 MPa/150 °C/10 min	5.6	300 °C/30 min	5.8	90.9	[86]	2020
10 wt.%FeCl_3_	3 MPa/300 °C/2 min	5.21	320 °C/10 min	5.45	130	[87]	2014
FeNi_2_S_4_	3 MPa/200 °C/1 min	4.7	300 °C/60 min	1.92	65.5	[88]	2022
5 wt.%FeOOH	3.2 MPa/200 °C/60 min	4.4	300 °C/60 min	5.5	128.6	[89]	2022
5 wt.%Fe/rGO	3 MPa/100 °C/8.3 min	0.5	250 °C/20 min	0.8	126.3	[84]	2020
5 wt.%Ni_3_Fe/rGO	3 MPa/100 °C/1.3 min	6	250 °C/20 min	4.8	59.3	[84]	2020
5 wt.%FeCoNi@GS	1.5 MPa/290 °C/1.65 min	6.01	290 °C/8.5 min	6.14	85.14	[85]	2020
10 wt.%CoFeB/CNTs	5 MPa/150 °C/10 min	6.2	300 °C/30 min	6.5	83.2	[86]	2020
10 wt.%Fe–Ni@3DG	5 MPa/300 °C/1.7 min	~6.2	300 °C/8.3 min	~5.2	83.8	[90]	2021
10 wt.%Ni_3_Fe/BC	3 MPa/150 °C/10 min	5.35	300 °C/7 min	6.48	102.01	[91]	2022
10 wt.%FeOOH NDs@G	3.2 MPa/200 °C/60 min	6.0	300 °C/60 min	6.6	125.04	[89]	2022
Ni/Fe_3_O_4_@MIL	3 MPa/150 °C/60 min	~5.42	350 °C/8 min	~4.8	97.94	[92]	2022

**Table 5:** Hydrogen storage performance of Mg/MgH_2_ system doped with titanium-based catalysts.

Catalysts	Hydrogen Absorption Conditions	Hydrogen Absorption Capacity (wt.%)	Dehydrogenation Conditions	Dehydrogenation Capacity (wt.%)	Dehydrogenation Ea (kJ/mol)	Ref.	Year
5 wt.%Ti	1.5 MPa/270 °C/15 min	4.3	320 °C	——	62.20	[63]	2013
7 wt.%Ti	1.2 MPa/270 °C/8 min	3.8	——	——	——	[96]	2014
10 wt.%TiFe	3 MPa/125 °C/60 min	5.3	300 °C/10 min	6.6	80.9	[97]	2021
10 wt.%TiMgVNi_3_	——	——	325 °C/10 min	5.19	94.4	[98]	2022
7 wt.%TiO_2_	1.2 MPa/270 °C/8 min	4.2	——	——	——	[96]	2014
7 wt.%TiCl_3_	1.2 MPa/270 °C/8 min	4.5	——	——	——	[96]	2014
7 wt.%TiF_3_	1.2 MPa/270 °C/8 min	5	300 °C/6 min	~2.7	——	[96]	2014
TiO_2_:QDs	225 °C/1.2 min	~5	300 °C/4.5 min	6.0	——	[99]	2021
10 wt.%(Ti_0.5_V_0.5_)_3_C_2_	5 MPa/120 °C/0.08 min	4.8	300 °C/7 min	6.0	77.3	[100]	2018
10 wt.%TiVO_3.5_	5 MPa/100 °C/0.08 min	3.9	250 °C/10 min	5.0	62.4	[101]	2018
5 wt.%F-Ti_3_C_2_T_x_	3 MPa/125 °C/20 min	~4.57	275 °C/16.7 min	~5.95	78.2	[102]	2022
5 wt.%E-F-Ti_3_C_2_T_x_	3 MPa/125 °C/20 min	~3.46	275 °C/16.7 min	~4.97	89.6	[102]	2022
Ti-Ni-Fe@Gr	1.5 MPa/300 °C/0.83 min	5.60	300 °C/8 min	5.70	——	[103]	2022
Ti@Gr	1.5 MPa/300 °C/0.83 min	5.30	300 °C/8 min	4.40	——	[103]	2022
TiH_2_@Gr	1.5 MPa/300 °C/2.5 min	5.64	300 °C/15 min	5.48	88.89	[104]	2020
TiO_2_@Gr	1.5 MPa/300 °C/2.5 min	5.59	300 °C/15 min	4.87	98.00	[104]	2020
fl-TiO_2_@C	150 °C/40 min	6.3	250 °C/7 min	6.0	67.10	[105]	2020
5 wt.%Ni/Ti_3_C_2_-WE	3 MPa/200 °C/0.83 min	5.6	275 °C/10 min	6.25	91.64	[106]	2021
5 wt.%6M-TiO_2_/FL-Ti_3_C_2_T_x_	3 MPa/175 °C/20 min	5.90	300 °C/10 min	5.98	96.7	[107]	2022

**Table 6:** Hydrogen storage performance of Mg/MgH_2_ system doped with vanadium-based catalysts.

Catalysts	Hydrogen Absorption Conditions	Hydrogen Absorption Capacity (wt.%)	Dehydrogenation Conditions	Dehydrogenation Capacity (wt.%)	Dehydrogenation Ea (kJ/mol)	Ref.	Year
5 wt.%V	1 MPa/300 °C/2.5 min	4.0	350 °C/5 min	3.0	——	[123]	2014
5 wt.%VC	1 MPa/300 °C/2.5 min	5.0	350 °C/5 min	5.5	63	[123]	2014
5 wt.%VCl_3_	1 MPa/300 °C/2.5 min	5.4	350 °C/5 min	6.0	47	[123]	2014
VO_2_(B)	350 °C/4.2 min	4.7	350 °C/2.2 min	4.9	60	[124]	2016
10 wt.% metallic glassy V_45_Zr_20_Ni_20_Cu_10_Al_3_Pd_2_	180 °C/1.67 min	5.35	180 °C/3 min	5.5	——	[125]	2018
10 wt.%VB_2_	——	——	300 °C/5 min	6.01	80.06	[126]	2021
10 wt.%V_2_O_5_	3 MPa/50 °C/30 min	3.59	260 °C/10 min	~5.0	——	[127]	2022
10 wt.%V_4_Nb_18_O_55_	3 MPa/50 °C/30 min	4.06	260 °C/10 min	~5.5	——	[127]	2022
10 wt.%VNbO	3 MPa/50 °C/30 min	4.34	260 °C/5 min	~6.0	78.2	[127]	2022
9 wt.%V_2_O_3_@C	5 MPa/150 °C/13.3 min	6.0	275 °C/10 min	6.0	70	[128]	2018

**Table 7:** Hydrogen storage performance of Mg/MgH_2_ system doped with manganese-based catalysts.

Catalysts	Hydrogen Absorption Conditions	Hydrogen Absorption Capacity (wt.%)	Dehydrogenation Conditions	Dehydrogenation Capacity (wt.%)	Dehydrogenation Ea (kJ/mol)	Ref.	Year
10 wt.%submicron-Mn	3 MPa/100 °C/30 min	3.0	300 °C/8 min	6.6	——	[130]	2020
10 wt.%nano-Mn	3 MPa/100 °C/30 min	3.3	300 °C/5 min	6.7	——	[131]	2021
10 wt.%submicron-LaNi_4.5_Mn_0.5_	3 MPa/150 °C/10 min	4.1	300 °C/6 min	6.6	——	[132]	2020
10 wt.%Mn_3_O_4_	3 MPa/100 °C/10 min	5.0	300 °C/8 min	6.8	——	[133]	2020
10 wt.%MnO	200 °C/20 min	~4.5	300 °C/60 min	~6.0	103.9	[134]	2021
MnS	100 °C/60 min	2.6	300 °C/30 min	4.6	93.7	[135]	2021
10 wt.%MnMoO_4_	3 MPa/150 °C/10 min	4.0	300 °C/10 min	6.03	109.9	[136]	2021
10 wt.%MnO@C	150 °C/10 min	~5.8	300 °C/10 min	~6.0	94.6	[134]	2021

## Data Availability

Not applicable.
